# Enhanced Solubilization and Biodegradation of HMW-PAHs in Water with a *Pseudomonas mosselii*-Released Biosurfactant

**DOI:** 10.3390/polym15234571

**Published:** 2023-11-29

**Authors:** Mingqian Xia, Shibin Wang, Bo Chen, Rongpeng Qiu, Gongduan Fan

**Affiliations:** College of Civil Engineering, Fuzhou University, Fuzhou 350116, China

**Keywords:** biosurfactant, *Pseudomonas mosselii*, HMW-PAHs, biodegradation, environmental stress

## Abstract

The treatment and reuse of wastewater are crucial for the effective utilization and protection of global water resources. Polycyclic aromatic hydrocarbons (PAHs), as one of the most common organic pollutants in industrial wastewater, are difficult to remove due to their relatively low solubility and bioavailability in the water environment. However, biosurfactants with both hydrophilic and hydrophobic groups are effective in overcoming these difficulties. Therefore, a biosurfactant-producing strain *Pseudomonas mosselii* MP-6 was isolated in this study to enhance the bioavailability and biodegradation of PAHs, especially high-molecular-weight PAHs (HMW-PAHs). FTIR and LC-MS analysis showed that the MP-6 surfactant belongs to rhamnolipids, a type of biopolymer, which can reduce the water surface tension from 73.20 mN/m to 30.61 mN/m at a critical micelle concentration (CMC = 93.17 mg/L). The enhanced solubilization and biodegradation of PAHs, particularly HMW-PAHs (when MP-6 was introduced), were also demonstrated in experiments. Furthermore, comprehensive environmental stress tolerance tests were conducted to confirm the robustness of the MP-6 biosurfactant, which signifies the potential adaptability and applicability of this biosurfactant in diverse environmental remediation scenarios. The results of this study, therefore, have significant implications for future applications in the treatment of wastewater containing HMW-PAHs, such as coking wastewater.

## 1. Introduction

Water resources are essential for life and human society. This is especially apparent in places where water shortages are posing great challenges to people’s daily life. The safe and efficient reuse of water, as a means to protect water resources, is thus crucial for sustainable societal development. Polycyclic aromatic hydrocarbons (PAHs), one of the most common persistent organic contaminants in industrial wastewater, road runoff, even reclaimed water, etc., have been the focus of many research projects in recent years [1,2]. Most of the research showed that low-molecular-weight polycyclic aromatic hydrocarbons (LMW-PAHs), containing two to three aromatic benzene rings, such as naphthalene (NAP) and phenanthrene (PHE), were easy to degrade with microorganisms. For example, Parab et al. applied a bacterial complex in membrane bioreactors to treat PAH-contaminated wastewater. The results showed that the bacterial complex was able to degrade a mixture of 99.9% NAP and 92.9% PHE (2000 mg/L) within 6 days [3]. Liu et al. isolated a new strain of *Pseudomonas aeruginosa* MPDS, which can degrade 100% of NAP (1000 mg/L) within 84 h and 40.3% of FLU (100 mg/L) within 72 h [4]. However, PAHs with four or more benzene rings, called high-molecular-weight polycyclic aromatic hydrocarbons (HMW-PAHs), including pyrene (PYR), benzo [a] pyrene (BaP), etc., have low solubility in water, which greatly limits their bioavailability, and makes it difficult to achieve a high biodegradation efficiency [5,6].

Surfactants, a kind of amphiphilic substances with both hydrophilic and hydrophobic molecular groups, are able to distribute at interfaces between fluid phases with different polarity and hydrogen bonds, such as those between oil and water, or air and water [7]. This characteristic can help reduce the surface and interfacial tension of PAHs/water, leading to the formation of a hydrocarbon-soluble or water-soluble microemulsion [7], which helps the biodegradation of PAHs. At present, most surfactants available on the market are chemical (synthetic) surfactants, such as non-ionic (Tween 20, Tween 80), cationic (dodecyl ethyl dimethyl ammonium bromide, hexadecyl trimethylammonium bromide), and anionic (sodium dodecyl sulfate) surfactants [8]. These surfactants can greatly increase the solubility of PAHs in soil, sediment, and water, enhancing their biodegradation efficiency. The bioremediation process assisted with the addition of these chemicals is thus called surfactant-enhanced bioremediation (SER) [9]. For example, the research of Gharibzadeh F. et al. showed that adding Tween 80 (5000 mg/L) increased the removal efficiency of PHE in an aqueous solution to 99%, which was 25% higher than the control [10]. However, despite their significant effect on the biodegradation of PAHs in water, synthetic surfactants also have drawbacks, such as high toxicity and low biodegradability, which can adversely affect our environment [11].

Meanwhile, there is another kind of surfactants from an alternative source, namely natural (biological) surfactants. They are usually secondary metabolites of microorganisms, produced during the logarithmic and stable phases in the growth curve. This class of surfactants can be roughly divided into glycolipids, lipoproteins or lipopeptides, phospholipids, fatty acids or natural lipids, polymeric surfactants, and particulate surfactants [12,13]. Compared to chemical (synthetic) surfactants, biosurfactants have the following advantages, making them a more viable option than their synthetic counterparts in the future: (1) good environmental compatibility, as they are relatively non-toxic and biodegradable [7]; (2) unique structural characteristics, which are crucial for the potential applications in industrial fields, ranging from biotechnology to environmental remediation; (3) better foaming ability, high selectivity and specific activity under hostile environmental conditions, including wide ranges of temperatures, pHs, and salinities; (4) synthesizability from renewable raw materials, etc. [7]. Currently, the known microbial genera that can produce surfactants include *Actinobacteria*, *Bacillus*, *Pseudomonas*, *Enterobacterium*, *Rhodococcus*, *Serratia*, *Streptococcus*, etc. [13,14]. Among numerous biosurfactants, rhamnolipids (secondary metabolites of *Pseudomonas*) have been widely studied and applied in various industrial and environmental applications [15].

With the help of biosurfactants, hydrocarbon molecules can be utilized by microorganisms after diffusion into the aqueous phase or directly absorbed by microorganisms from micelles. The process of microbial uptake of hydrocarbons from surfactant micelles can be divided into three different stages [16,17]. In the first stage, dissolved substrate micelles are transported to the vicinity of cells; then, material exchange occurs between the substrate filled micelles and the semi micelle layer of surfactant molecules formed around the cell. Finally, the substrate is transferred from the semi micelle layer to the bacterial cell. Studies have shown that the improvement in the biodegradation efficiency of PAHs using biosurfactants can exceed that of chemical surfactants. For example, Wang et al. [18]’s research showed that PAHs contaminated soil treated with a biosurfactant rhamnolipid achieved the highest biodegradation rate of 95%, compared with chemical surfactants comprising Tween 80 (92%) and SDS (90%). In addition, Lamichhane et al. [17]’s research found that saponins, a typical kind of plant-derived biosurfactants, have a higher solubilization effect on BaP than Tween 80, Brij58, and TX100 (chemical surfactants).

In this study, a biosurfactant-producing strain *Pseudomonas mosselii* MP-6 was isolated, and this biosurfactant was used to enhance the solubilization and biodegradation efficiencies of four selected PAHs: two LMW-PAHs (naphthalene (NAP), phenanthrene (PHE)) and two HMW-PAHs (pyrene (PYR), benzo[a]pyrene (BaP)). To the best of our knowledge, there is currently no report on the secretion and extraction of biosurfactants produced with *P. mosselii* for this purpose. Experiments were also performed to determine the optimal growth/fermentation conditions for strains/biosurfactants, and their tolerance to extreme environmental conditions (various pHs, temperatures, NaCl concentrations, etc.). In addition, the acid precipitation method, FTIR, and LC-MS were used to extract and determine the chemical structures of biosurfactants. Last but not least, solubilization characteristics of the biosurfactants on single and mixed substrates of NAP, PHE, PYR, and BaP and their effects on the biodegradation of HMW-PAHs were also studied.

## 2. Materials and Methods

### 2.1. Screening of Biosurfactant-Producing Strains

Strains previously isolated from activated sludge, stored in the laboratory under −80 °C, were selected and their ability to produce biosurfactants was evaluated using the combination of oil spreading method, drop collapse test, surface tension measurement, and emulsification stability test (E24). The details for each test can be found in Text S1.

### 2.2. Identification of Bacterial Strains with 16S rDNA Sequencing

Bacterial strains stored at −80 °C were retrieved through streaking plate method to ensure their purity. They were then inoculated into the LB broth to grow at 30 °C and 130 rpm for 12–48 h, depending on the bacterial growth. DNA extraction kit (column type) from TIANGEN (Beijing, China) was used to extract the bacterial DNA, which was subsequently sent to Sangon Biotech Co., (Shanghai, China) for 16S rDNA sequencing. DNASTAR 11.0 was then used to assemble and organize the obtained sequences. Finally, with MEGA-X, a phylogenetic tree of the bacterial 16S rDNA was constructed using the neighbor-joining method.

### 2.3. SEM Observation of the Bacterial Strains

SEM was used to observe the morphology of the selected strains. The specific sample preparation method was modified from Nalvothula et al. [19] and can be found in Text S2.

### 2.4. Optimal Growth Conditions of the Strains

#### 2.4.1. Growth Curve of the Strains

The target strain growing to the logarithmic phase was collected, and OD_600_ measurements were adjusted to 0.5 before its inoculation (10%) to the LB broth for the measurement of the growth curve (under 30 °C, 130 rpm for 48 h). Samples were taken at different time points (every 2 h during the first 24 h, and every 4 h during the subsequent 24 h) for OD_600_ measurements using a spectrophotometer. Unless otherwise specified, the experiments in this study were performed in triplicate.

#### 2.4.2. Tolerance to Extreme Environmental Conditions

To investigate the effects of initial salt concentrations, pH values, and heavy metal concentrations on the growth of strain, different LB liquid media were prepared with the addition of varying amounts of NaCl, HCl/NaOH, NiCl_2_, Cd(NO_3_)_2_, Pb(NO_3_)_2_, and K_2_Cr_2_O_7_. Specifically, the following gradients were used: NaCl concentrations of 10, 20, 30, 40, 50, 60, 80, and 100 g/L; pH values of 4, 5, 6, 7, 8, 9, and 10; Ni^2+^, Cd^2+^, Pb^2+^, and Cr^6+^ concentrations of 0, 10, 20, 30, 40, 60, 80, and 100 mg/L. All the media were sterilized under 121 °C for 20 min before use. Growth curve measurement method can be found in Section 2.4.1.

### 2.5. Experimental Study on Biosurfactant Production with Pseudomonas mosselii MP-6

#### 2.5.1. Optimal Fermentation Conditions for Biosurfactant Production

##### Carbon Source

To investigate the optimal carbon source for biosurfactant production with *P. mosselii* MP-6, various carbon sources were added to a minimal salt medium (MSM) at concentrations of 20 g/L glucose, 20 g/L sodium citrate, 20 g/L sodium acetate, 20 g/L sodium oxalate, 2 mL/100 mL of gasoline, and 2 mL/100 mL of rapeseed oil, respectively. The MSM used in this study contained 3.7 g/L KH_2_PO_4_, 5.2 g/L K_2_HPO_4_·3H_2_O, 2 g/L NH_4_Cl, 1 g/L Na_2_SO_4_, 0.1 g/L MgSO_4_, and 1 mL trace metals’ solution (0.3 g/L FeCl_2_·4H_2_O, 0.038 g/L CoCl_2_·6H_2_O, 0.02 g/L MnCl_2_·4H_2_O, 0.014 g/L ZnCl_2_, 0.0124 g/L H_3_BO_3_, 0.04 g/L Na_2_MoO_4_·2H_2_O, 0.0034 g/L CuCl_2_·2H_2_O). *P. mosselii* MP-6 was inoculated into different media at a 10% inoculum rate and incubated under 30 °C, 130 rpm. After 72 h, the samples were collected, and the emulsification stability (E24) as well as the surface tension of each sample were measured. 

##### Cultivation Time

*P. mosselii* MP-6 was inoculated into the MSM with 20 g/L sodium citrate at a 10% rate and then incubated on a shaker at 30 °C, 130 rpm. Samples were taken at 0, 24, 48, 72, 96, 120, 144, and 168 h for OD_600_ measurements. The cell-free supernatants were also sampled for measurement of their emulsification stability (E24) and surface tension. 

#### 2.5.2. Extraction of the Biosurfactant

Acid precipitation and organic solvent extraction were used to the extract biosurfactant produced with *P. mosselii* MP-6. Initially, the strain was inoculated into the MSM with 20 g/L sodium citrate at a 10% rate and incubated under 30 °C, 130 rpm, for 7 days. Then, the bacterial culture was harvested and centrifuged at 8000 rpm for 20 min to remove the cells. The cell-free supernatant was collected, with its pH adjusted to 2.0 using 2 mol/L hydrochloric acid, and stored at 4 °C for 12 h. The solution was then centrifuged again at 8000 rpm for 20 min, when the supernatant was discarded. The precipitate obtained was redissolved in a small amount of ultrapure water, and the pH was adjusted to 7.0 using 2 mol/L sodium hydroxide. The sample was then placed in a freezer at −20 °C for 12 h, then freeze-dried in a vacuum freeze dryer for 24 h. The resulting product is a crude extract biosurfactant produced with *P. mosselii* MP-6. Then, the organic solvent extraction method was used to purify the crude extract further. A certain volume of a mixed organic solvent (V_chloroform_:V_methanol_ = 2:1) was used to dissolve the crude extract biosurfactant through ultrasonication. The insoluble substances were then removed with centrifugation at 8000 rpm for 20 min. The resulting supernatant was evaporated to dryness using N_2_ gas [20]. After complete evaporation, the remaining substance was a purified biosurfactant.

#### 2.5.3. Physicochemical Properties of the Biosurfactant

##### Chemical Structure of the Biosurfactant

FTIR and LC-MS were used to determine the chemical structure of the biosurfactant. For the FTIR measurement, the purified biosurfactant was dissolved in 1 mL methanol and then sent for functional groups analysis between the wavenumber range of 4000 and 400 cm^−1^ [7]. For the LC-MS measurement, the purified biosurfactant was dissolved in 1 mL methanol and then characterized using HPLC-QTOF-MS (1260–6520 model, Agilent, CA, USA). HPLC-MS analysis was performed in positive ion mode using an Agilent Zorbax SB-C18 column (4.6 mm × 150 mm, 5 μm). The instrument was equipped with an electrospray ionization (ESI) source with pneumatic assistance. Nitrogen gas was used as a nebulizing gas at a flow rate of 10 L/min, a temperature of 350 °C, and a capillary voltage of 3.5 kV. The sample was introduced into the heated ESI (H-ESI) ion source using the flow injection analysis (FIA) method. The mobile phase consisted of 0.1% formic acid/100% methanol (75:25, *v*/*v*) at a flow rate of 0.8 mL/min. The column temperature was maintained at 30 °C, and the injection volume was 20 μL. A complete scan data was obtained by scanning the *m*/*z* range of 50 to 1000, with the ion spray voltage set at 40 V [7].

##### Determination of the Critical Micelle Concentration (CMC) of the Biosurfactant

The biosurfactant was dissolved in ultrapure water to prepare solutions with concentrations of 10, 20, 30, 40, 50, 60, 80, 100, 120, 140, 160, 180, and 200 mg/L. The surface tension values of these solutions were measured and plotted in a graph against an x-axis of the logarithm (base 10) values of the biosurfactant’s concentrations. The inflection point on the graph represents the critical micelle concentration (CMC) of the biosurfactant produced with *P. mosselii* MP-6.

#### 2.5.4. Tolerance of the Biosurfactant to Extreme Environmental Conditions

*P. mosselii* MP-6 was inoculated into the MSM containing 20 g/L sodium citrate at a 10% (*v*/*v*) inoculation rate and incubated on a shaker under 30 °C, 130 rpm, for 168 h. The stability of the produced biosurfactant was measured under different environmental conditions with a wide range of temperatures (4, 10, 20, 30, 40, 50, 60, 70, 80, 90, and 100 °C), NaCl concentrations (0, 10, 20, 30, 40, 50, 60, 80, and 100 g/L), pH (4, 5, 6, 7, 8, 9, and 10), and heavy metals (Ni^2+^, Cd^2+^, Pb^2+^, and Cr^6+^); detailed information of the experiment design can be found in Text S3.

### 2.6. Solubilization and Biodegradation Promotion Effects of the Biosurfactant on PAHs

#### 2.6.1. Promotion of the Solubilization of PAHs with the Biosurfactant

To investigate the effects of the biosurfactant on the solubilization of PAHs, a purified biosurfactant was dissolved in sterilized ultrapure water to obtain solutions with different concentrations, including 0, 0.5, 1, 2, 3, 4, and 5 CMC. Excess NAP, PHE, PYR, BaP, or a mixture of these compounds, were each added to a 100 mL conical flask to act as the substrate. Then, 20 mL of purified biosurfactant solution was added to the flask, which was subsequently placed in a constant temperature incubator at 30 °C, 130 rpm, for 48 h. Undissolved PAHs were then removed with centrifugation at 5000 rpm for 20 min. The amount of dissolved PAHs were determined using HPLC; detailed information can be found in Text S4.

#### 2.6.2. Promotion of the Biodegradation of PAHs with the Biosurfactant

To investigate the influence of adding biosurfactants on the biodegradation of PAHs, purified biosurfactants were added to the MSM to obtain solutions with varying concentrations of biosurfactants: 0, 1, 2, 3, 4, and 5 CMC. Subsequently, PAH stock solutions (10 mg/L NAP, 10 mg/L PHE, 10 mg/L PYR, 10 mg/L BaP) were added to conical flasks and placed in a laminar flow hood until the acetonitrile completely evaporated. The pre-existing degradation strain *Pseudomonas citronellolis* MP-7 was inoculated into the degradation medium with different CMC at a 10% (*v*/*v*) inoculation rate and incubated in a constant temperature incubator at 30 °C, 130 rpm, for 7 days. Then, the samples were taken out for HPLC analysis; detailed information can be found in Text S4.

## 3. Results and Discussion

### 3.1. Screening of Biosurfactant-Producing Strains

A biosurfactant-producing strain MP-6 was screened out from existing laboratory strains through the oil spreading method, drop collapse test, surface tension measurement, and emulsification stability test (E24); the results can be found in Figure S1 and Table S1. It can be observed that among the strains showing positive results in the drop collapse assay, only the MP-6 strain exhibited a complete collapse (Figure S1a). The order of the diameters of oil clearing zones was as follows: MP-6 > MP-8 > MP-1 > MP-10. Moreover, MP-6 demonstrated the largest clearing zone diameter (21.3 ± 1.1 mm). In the emulsification stability (E24) test, the order of emulsion layer heights was MP-12 > MP-3 > MP-6 > MP-14 > MP-8. Regarding the surface tension measurement of the supernatants, the order of capabilities to reduce liquid surface tensions was MP-6 > MP-10 > MP-9 > MP-8. In summary, the MP-6 strain showed the best performance in the screening tests and was ultimately selected as the research subject for biosurfactant production.

### 3.2. Physiological Characteristics of P. mosselii MP-6

#### 3.2.1. Identification of the Strain

To identify the MP-6 strain, 16S rDNA sequencing results were submitted to NCBI for a BLAST search, and the homology was compared with the 16S rDNA sequences previously uploaded to the system. The results showed that MP-6 belongs to *Pseudomonas mosselii*, and the 16S rDNA sequence of *P. mosselii* MP-6 has also been uploaded to GenBank (accession number OP954494).

#### 3.2.2. SEM Observation of the Strain

**Figure 1:**
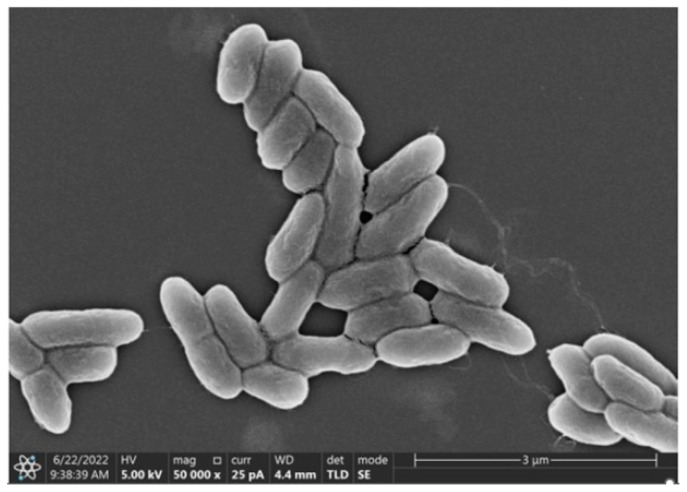
SEM image of *P. mosselii* MP-6 (50,000× magnification).

Scanning electron microscopy (SEM) was used to observe the morphology of *P. mosselii* MP-6, and the results are shown in Figure 1. It can be seen that MP-6 has a smooth surface and rod-shaped cells with sizes ranging from approximately 1 to 2 μm.

#### 3.2.3. *P. mosselii* MP-6 Growth under Different Environmental Conditions

The growth curve of *P. mosselii* MP-6 in LB broth under 30 °C is shown in Figure S3a. From the figure, it can be observed that *P. mosselii* MP-6 entered the logarithmic growth phase at around 2 h, followed by a stable phase from 24 to 36 h. Meanwhile, the correlations of the OD_600_ measurements with the total bacterial count of *P. mosselii* MP-6 can be seen in Figure S3b. More specifically, a linear relationship is evident, which can be expressed as follows: total bacterial count = (4.37112 × OD_600_ − 0.4282) × 10^7^ CFU/mL.

The growth curves of *P. mosselii* MP-6 under environmental conditions comprising pH (4–10), NaCl (0–100 g/L), Ni^2+^ (0–100 mg/L), Pb^2+^ (0–100 mg/L), Cr^6+^ (0–100 mg/L), and Cd^2+^ (0–100 mg/L) are shown in Figure S4. From the figure, it can be observed that *P. mosselii* MP-6 exhibited good tolerance to pH variations (4–10), and also relatively high tolerance to NaCl stresses. It could grow within the range of 0–80 g/L NaCl. However, significant growth inhibition occurred when the NaCl concentration exceeded 40 g/L (the salt concentration in seawater is approximately 35 g/L). Furthermore, from Figure S4c–f, it can be found that *P. mosselii* MP-6 was capable of growing under different concentrations of heavy metals: Ni^2+^, Pb^2+^, Cr^6+^, and Cd^2+^ (0–100 mg/L). Specifically, *P. mosselii* MP-6 exhibited good tolerance to Ni^2+^ and Pb^2+^ within the range of 0–100 mg/L. However, for Cr^6+^ and Cd^2+^, when the concentrations exceeded 80 mg/L, the growth of *P. mosselii* MP-6 was significantly inhibited.

### 3.3. Optimal Fermentation Conditions for Biosurfactant Production

#### 3.3.1. Carbon Source

**Figure 2:**
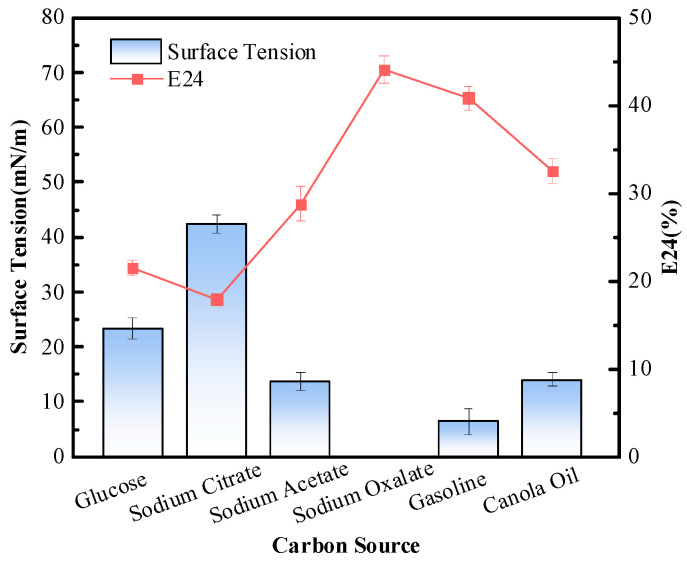
Effects of different carbon sources on biosurfactant production with *P. mosselii* MP-6.

A carbon source provides energy for bacterial growth and reproduction while also influencing the yield of their metabolites [21]. In this study, the biosurfactant production of *P. mosselii* MP-6 was measured with supplies of different carbon sources, and the results are shown in Figure 2. From the figure, it can be observed that after 72 h of cultivation under the same conditions, *P. mosselii* MP-6 had the highest biosurfactant production in a medium with 20 g/L sodium citrate as the carbon source; moreover, the culture supernatant was able to reduce the surface tension to 28.7 mN/m and exhibited an emulsification stability (E24) of 26.5% towards gasoline. Glucose, on the other hand, resulted in a lower biosurfactant production with a reduction in surface tension to 34.3 mN/m and an emulsification stability of 14.6% towards gasoline. Meanwhile, when sodium oxalate was used as the carbon source, *P. mosselii* MP-6 produced almost no biosurfactants. Therefore, among the tested carbon sources, the optimal carbon source for *P. mosselii* MP-6 biosurfactant production was sodium citrate, followed by glucose, while sodium oxalate was not suitable for MP-6 biosurfactant production.

#### 3.3.2. Cultivation Time

**Figure 3:**
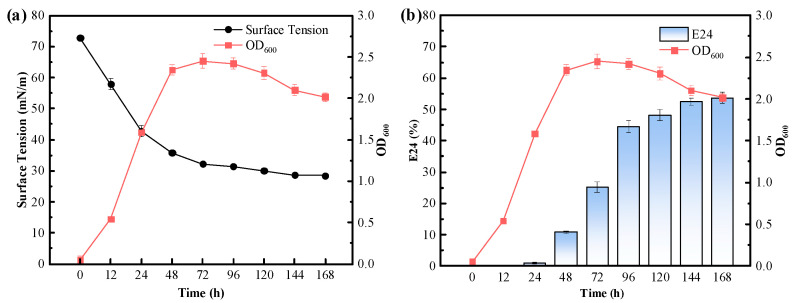
(**a**) Emulsifying index E24 and (**b**) surface tension of *P. mosselii* MP-6 culture supernatant at different incubation times.

To determine the effect of cultivation time on the biosurfactant production of *P. mosselii* MP-6, the growth curve, surface tension, and emulsification index (E24) of the bacterial culture supernatant in a sodium citrate fermentation medium were measured, and the results are shown in Figure 3. From the figure, it can be observed that as the cultivation time increased, the emulsification index (E24) of the bacterial culture supernatant continuously increased, while the surface tension decreased. The strain entered the logarithmic phase after 2 days of inoculation, followed by a stable phase after 3 days, during which the highest number of viable cells capable of producing a large amount of secondary metabolites were present, including biosurfactants. By the seventh day, the concentration of the biosurfactant in the culture supernatant reached its highest value. The surface tension of the supernatant was reduced to 28.5 mN/m, and the emulsification index (E24) reached a maximum value of 53.70 ± 1.85%. 

According to the experimental results, the highest yield of biosurfactant production with MP-6 can be achieved after 7 days of cultivation in a fermentation medium containing 20 g/L sodium citrate as the carbon source. A total of 1.82 ± 0.21 g/L of a biosurfactant crude extract was obtained through the acid precipitation method; subsequently, a 0.81 ± 0.11 g/L purified biosurfactant was obtained using organic solvents.

### 3.4. Physiochemical Properties of the Biosurfactant

#### 3.4.1. Chemical Structure of the Biosurfactant

FTIR analysis was performed to explore the functional groups of the biosurfactant produced with *P. mosselii* MP-6, and the results are shown in Figure S5. From the graph, it can be observed that there are no absorption peaks at 3422 cm^−1^, 3246 cm^−1^, and 1552 cm^−1^, indicating the absence of N–H bonds and amino groups [22]. In addition, a broad peak was observed at 3354 cm^−1^, corresponding to O–H groups. Meanwhile, the sharp peaks appearing at 2944 cm^−1^ and 2833 cm^−1^ are characteristic peaks of C–H (CH_2_–CH_3_) groups. Finally, the presence of COO– C=O, and C–O–C functional groups were, respectively, confirmed by the peaks at 1660 cm^−1^, 1413 cm^−1^, and 1032 cm^−1^ [7]. Based on the results of FTIR analysis, the biosurfactant exhibited functional group characteristics of both carbohydrates and lipids [13]. Therefore, it was speculated that the biosurfactant produced with *P. mosselii* MP-6 belonged to the class of glycolipid biosurfactants.

The purified biosurfactant was also characterized with LC-MS, and the results are shown in Figure S6. From Figure S6a, it can be seen that the peak at 3–4 min is the characteristic peak of the solvent methanol, while the peaks appearing at 6–7 min are the characteristic peaks of the biosurfactant. Figure S6b showed the mass spectrometry results of the biosurfactant, with strong mass spectral peaks at 701.430, 583.392, and 333.180, indicating the presence of monorhamnolipid Rha-C12-C12 (*m*/*z* = 583.392), dirhamnolipid Rha-Rha-C10-C12 (*m*/*z* = 701.430), and monorhamnolipid Rha-C10 (*m*/*z* = 333.180) [23]. This is similar to the results of Zhao et al. [24], where the metabolites of *Pseudomonas* were analyzed using HPLC-MS and the coexistence of monorhamnolipids and dirhamnolipids was also revealed. Therefore, the biosurfactant produced with *P. mosselii* MP-6 belongs to the rhamnolipid class, comprising a mixture of several congeners of monorhamnolipids and dirhamnolipids. 

#### 3.4.2. Critical Micelle Concentrations (CMCs) of Biosurfactants

The critical micelle concentration (CMC) is one of the most important indicators for evaluating the performance of surfactants. As can be seen from Figure S7, with the increase in biosurfactant concentrations, the surface tension gradually decreased; however, the rate of change during this process can vary. When the added biosurfactant concentrations were below a certain point, the rates of surface tension reduction were relatively fast. However, when the concentration went above that point, a slower rate of surface tension reduction was observed. This inflection point corresponds to the concentration at which the biosurfactant produced with *P. mosselii* MP-6 began to form micelles and is known as the CMC. In this study, the CMC value of the biosurfactant was determined to be 93.17 mg/L. At this concentration, the biosurfactant was able to reduce the water surface tension from 73.20 mN/m to 30.61 mN/m. The CMC values of biosurfactants typically range from 10 to 540 mg/L, due to the differences in the degree of purification and their composition [25]. For example, Patowary et al. [26] purified a bacterial biosurfactant with a CMC of 56 mg/L, which could reduce water surface tension from 72.80 mN/m to 29.6 mN/m. Bharali et al. [27] reported a *P. aeruginosa* JBK1 strain, which could produce biosurfactants with a CMC of 540 mg/L. In comparison, the biosurfactant produced with *P. mosselii* MP-6 in this study exhibited a relatively low CMC (CMC of SDS, a typical synthetic surfactant, is 2 g/L) [25], indicating that it can be used as a potential additive for HMW-PAHs bioremediation enhancement.

#### 3.4.3. Tolerance of the Biosurfactant to Environmental Stresses

**Figure 4:**
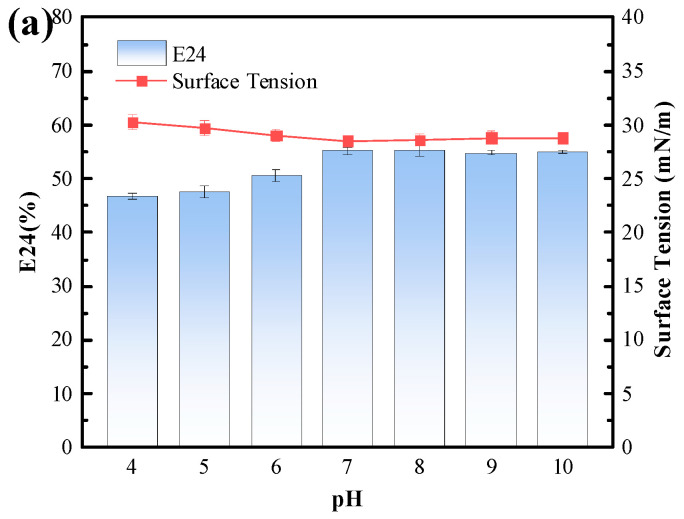

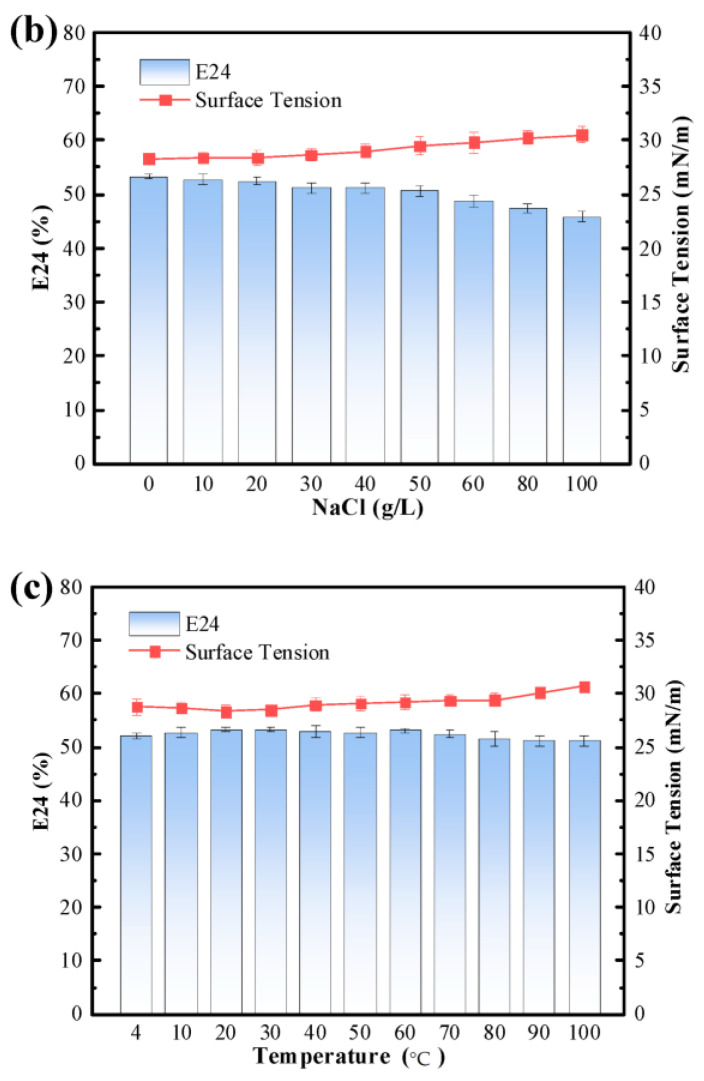
Biosurfactant tolerance to different (**a**) pH values, (**b**) NaCl concentrations, and (**c**) temperatures.

**Figure 5:**
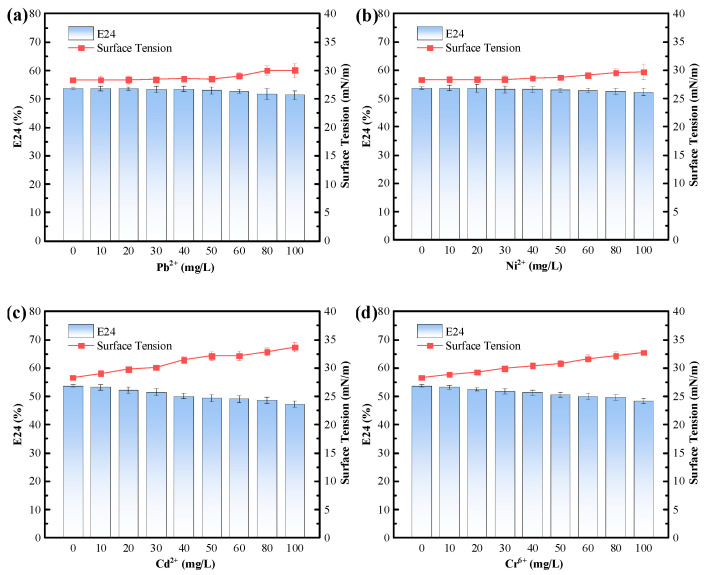
Biosurfactant tolerance to different heavy metals: (**a**) Pb^2+^, (**b**) Ni^2+^, (**c**) Cd^2+^, and (**d**) Cr^6+^.

The tolerance of the biosurfactant to environmental stressors, including pHs (4–10), temperatures (4 to 100 °C), NaCl concentrations (0 to 100 g/L), and heavy metals (Ni^2+^, Cd^2+^, Pb^2+^, and Cr^6+^), was tested; the results are shown in Figure 4 and Figure 5. It can be seen from the figures that the biosurfactant had a good tolerance to pH variations (from 4–10), with a slightly lower tolerance to acidic conditions. When the pH was reduced to 4, the emulsification index of the biosurfactant decreased to 46.78%, and the liquid surface tension increased to 30.29 mN/m. For NaCl concentrations, the biosurfactant exhibited an excellent tolerance to NaCl concentrations ranging from 0 to 20 g/L, with the emulsification index maintained at 52.50–53.33%, and the liquid surface tension maintained at 28.33–28.38 mN/m. As the NaCl concentration increased from 0 g/L to 50 g/L, the emulsification index (E24) of the biosurfactant gradually decreased but still remained above 50%, while the variation in liquid surface tension was minimal. When the NaCl concentration reached 100 g/L, the emulsification index of the biosurfactant only dropped to 45.8%, while the liquid surface tension increased to 30.52 mN/m, demonstrating the excellent stability of the biosurfactant in the presence of salt ions. For temperature variations, there were no significant changes to the emulsification index and liquid surface tension of the biosurfactant from 4 to 100 °C, although it is worth noting that the optimal temperature range for the biosurfactant was 20–40 °C. As the temperature increased to more than 40 °C, the emulsification index of the biosurfactant gradually decreased, and the liquid surface tension gradually increased. 

The biosurfactant tolerance to Pb^2+^, Ni^2+^, Cd^2+^, and Cr^6+^ (0–100 mg/L) is shown in Figure 5. From Figure 5a,b, it can be seen that the biosurfactant had a good tolerance to Pb^2+^ and Ni^2+^ at concentrations from 0 to 100 mg/L, with little effect on its emulsification index and liquid surface tension. However, as the concentration of Cd^2+^ increased from 0 to 100 mg/L, the emulsification index of the biosurfactant decreased from 53.24% to 47.33%, and the liquid surface tension increased from 29.06 mN/m to 33.72 mN/m. Similarly, with an increase in the Cr^6+^ concentrations from 0 to 100 mg/L, the emulsification index (E24) decreased from 53.73% to 48.33%, and the liquid surface tension increased from 28.33 mN/m to 32.72 mN/m. Although high concentrations of Cd^2+^ and Cr^6+^ had some effects on the properties of the biosurfactant, it still remained largely stable. Therefore, the biosurfactants produced with *P. mosselii* MP-6 had a good tolerance to Pb^2+^, Ni^2+^, Cd^2+^, and Cr^6+^ stresses ranging from 0 to 100 mg/L.

### 3.5. Biosurfactant Effects on the Solubilization and Biodegradation of PAHs 

#### 3.5.1. Biosurfactant Effects on the Solubilization of PAHs 

**Figure 6:**
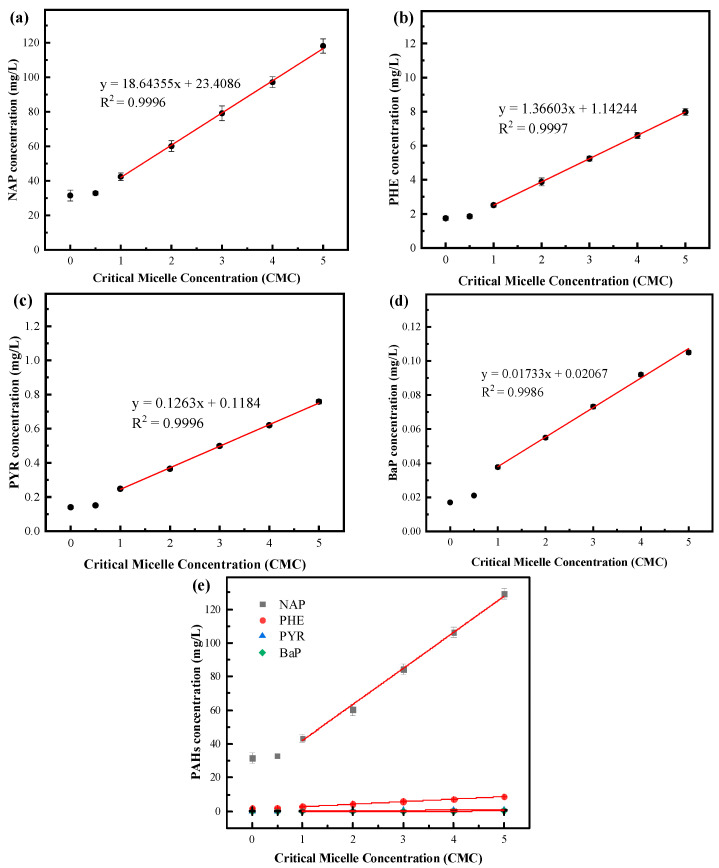
Effects of the biosurfactant on the solubilization of (**a**) NAP, (**b**) PHE, (**c**) PYR, (**d**) BaP, and (**e**) the mixed PAHs.

PAHs, especially HMW-PAHs, have high octanol–water partition coefficients and are practically insoluble in water; it is, therefore, generally believed that microorganisms cannot directly utilize these hydrophobic pollutants [28]. However, the addition of surfactant-like substances can effectively increase the solubility of PAHs in water and improve their mass transfer efficiencies between microorganisms, thereby enhancing microbial degradation efficiencies towards PAHs. Considering the above, this study selected four PAHs (NAP, a two-ring compound; PHE, a three-ring compound; PYR, a four-ring compound; BaP, a five-ring compound; chemical structures are shown in Figure S8) with relatively high concentrations in wastewater and different numbers of rings as the research objects. The effects and patterns of the solubilization of individual and mixed PAHs with varying concentrations of the biosurfactant were investigated, and the results are shown in Figure 6. Figure 6a–d presented the solubilization effects of the biosurfactant on individual substrates of NAP, PHE, PYR, and BaP, respectively. From the figures, it can be observed that the apparent solubility of NAP, PHE, PYR, and BaP increased with increasing concentrations of the biosurfactant (when concentrations were above 1 CMC). Within the range of 1–5 CMC, a linear relationship was observed between the concentrations of the biosurfactant and the solubility of PAHs. This might be because when the concentration of the biosurfactant reached 1 CMC and the free biosurfactant molecules in the solution started to aggregate and form micelles, which allowed PAHs to enter the hydrophobic cores of these micelles and thereby increased their solubility in the aqueous solution. As the concentration of the surfactants continued to increase, more PAHs could enter the hydrophobic cores, further enhancing their solubility in water [25]. Additionally, as shown in Figure 6e, when a mixture of NAP, PHE, PYR, and BaP was present, adding the biosurfactant did not only affect the solubility of each individual compound but also exhibited a synergistic solubilization effect on them. Similar results were also obtained by Liang et al. [29], where the solubility of the mixture of PHE and PYR increased by 15.38% and 18.19% (compared to the solubility of the individual compounds), respectively, when Triton X-100 was added to the solution. This may be due to the solubilization of low-molecular-weight PAHs, which reduced the interfacial tension and increased the volume of hydrophobic centers, collectively promoting the solubility of the remaining PAHs [25]. From the experiment results, the biosurfactant produced with *P. mosselii* MP-6 demonstrated promising effects on enhancing the solubilization of individual and mixed PAHs.

#### 3.5.2. Biosurfactant Effects on the Biodegradation of PAHs 

**Figure 7:**
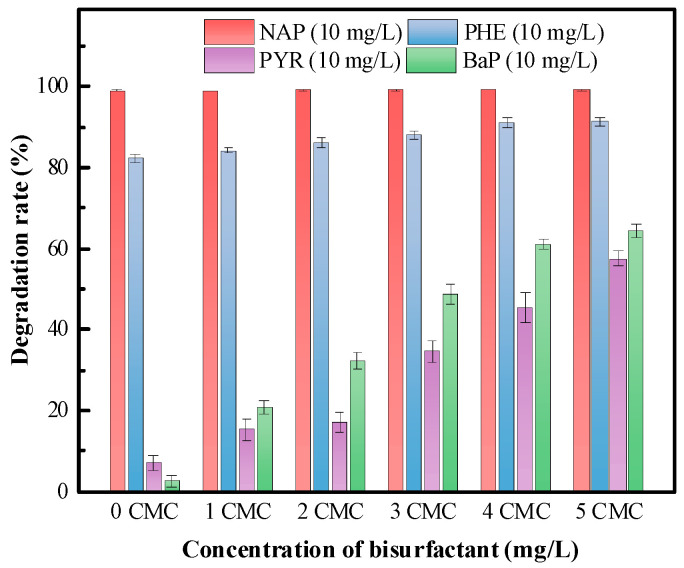
Effects of different concentrations of the biosurfactant on the biodegradation of PAHs.

To investigate the influence of the biosurfactant on the biodegradation of PAHs, a purified biosurfactant produced with *P. mosselii* MP-6 was added to the degradation system with PAHs and a degrading bacteria *P. citronellolis* strain MP-7; the results are shown in Figure 7. It can be observed from the figure that different concentrations of biosurfactants had varying degrees of impact on the degradation of PAHs. When the added biosurfactant concentration reached 5 CMC, *P. citronellolis* MP-7 achieved degradation rates of 100%, 91.36%, 57.56%, and 64.32% for NAP (10 mg/L), PHE (10 mg/L), PYR (10 mg/L), and BaP (10 mg/L), respectively, within 5 days under 30 °C. These degradation rates were improved by 0.24%, 8.97%, 50.38%, and 61.81%, respectively, compared to the cases without adding biosurfactants. Similar findings were reported by Parthipan et al. [30], who observed a 32.4% increase in the degradation rate of mixed PAHs (ANT, PYR, BaP) by a mixed microbial consortium with the addition of 5 mg/L lipopeptide biosurfactants. The heightened degradation efficiency in HMW-PAHs, with the addition of biosurfactants, might be attributed to the original low solubility or even insolubility of HMW-PAHs in water. As the biosurfactants increased their solubility, the mass transfer between microorganisms and pollutants was also significantly enhanced, and the biodegradation efficiency of PAHs was improved as a result [31]. As another example, Bezza et al. [32] used lipopeptide biosurfactants (consisted of surfactins, iturins, and fengycins) produced with *Bacillus cereus* SPL-4 to enhance the removal rates of PAHs in contaminated soil. The results showed that in microcosms supplemented with 0.2 and 0.6% (*w*/*w*) lipopeptide, the removal rates of 22.2% of four-ring and 29.5% of five- and six-ring PAHs as well as 35.1% of four-ring and 53.3% of five- and six-ring PAHs were enhanced, respectively, compared to the surfactant-free control after 64 days of treatment. There was no significant change in the degradation rates of LMW-PAHs with/without the presence of a surfactant. This is similar to the results found in this study, in which it was found that the biosurfactants produced with *P. mosselii* MP-6 significantly enhanced the degradation rates of all four PAHs, but with a much more pronounced effect on HMW-PAHs (PYR and BaP), demonstrating the potential of adding biosurfactants as a means to enhance PAHs bioremediation.

## 4. Conclusions

In this study, a biosurfactant-producing strain *P. mosselii* MP-6 was screened through the drop collapse test, oil spreading test, surface tension determination, and emulsification stability (E24) measurement. *P. mosselii* MP-6 had a good tolerance to environmental stressors including pH variations (4 to 10), NaCl concentrations (0 to 100 g/L), and heavy metals (such as Ni^2+^, Pb^2+^, Cr^6+^, and Cd^2+^). The optimal production of the biosurfactant produced with *P. mosselii* MP-6 was achieved when using 20 g/L sodium citrate as the carbon source and a fermentation cultivation period of 7 d. Acid precipitation and organic solvent extraction methods were employed to extract and purify the biosurfactant in the culture supernatant, resulting in a crude biosurfactant yield of 1.82 ± 0.21 g/L and a purified biosurfactant yield of 0.81 ± 0.11 g/L. FTIR and LC-MS analyses confirmed that the biosurfactant produced with MP-6 belongs to the rhamnolipid class. When the concentration of the biosurfactant reached a critical micelle concentration (CMC) of 93.17 mg/L, water surface tension could be reduced from 73.20 mN/m to 30.61 mN/m. Environmental stress tolerance tests showed that the biosurfactant had a high stability and could tolerate temperatures ranging from 4 to 100 °C, pH levels from 4 to 10, NaCl concentrations from 0 to 100 g/L, and was also resistant to heavy metal stresses. Adding this kind of biosurfactant at a concentration above the CMC can effectively increase the solubility of PAHs, especially HMW-PAHs. Moreover, a synergistic solubilization effect was observed for the mixed PAHs. When the added biosurfactant concentration reached 5 CMC, the biodegradation rates of the mixed NAP (10 mg/L), PHE (10 mg/L), PYR (10 mg/L), and BaP (10 mg/L) could be increased to 100%, 91.36%, 57.56%, and 64.32%, respectively, in 5 d under 30 °C. Moreover, the degradation rates were improved by 0.24%, 8.97%, 50.38%, and 61.81%, respectively, compared to the controls. In conclusion, the findings of this study provide a theoretical basis for using biosurfactants to enhance the treatment of wastewater containing PAHs, especially HMW-PAHs.

## Data Availability

Data are contained within the article and Supplementary Materials.

## Supplementary Materials

The following supporting information can be downloaded at: https://www.mdpi.com/article/10.3390/polym15234571/s1, Text S1. Screening for biosurfactant-producing strains; Text S2. SEM observation of cells; Text S3. Biosurfactant tolerance to extreme environmental conditions; Text S4. HPLC determination of PAHs; Figure S1. (a) Drop collapse results and (b) emulsification stability (E24) test of the bacterial supernatants; Figure S2. Fermentation culture of *P. mosselii* MP-6 and extraction process of the biosurfactant; Figure S3. (a) Growth curve of *P. mosselii* MP-6 in LB broth under 30 °C; (b) the relationship between optical density (OD_600_) and total bacterial count of *P. mosselii* MP-6; Figure S4. The growth curves of *P. mosselii* MP-6 under different (a) pH values; (b) NaCl concentrations, and heavy metals concentrations: (c) Ni^2+^, (d) Pb^2+^, (e) Cd^2+^, and (f) Cr^6+^; Figure S5. FTIR spectroscopy of the biosurfactant produced with *P. mosselii* MP-6; Figure S6. LC-MS results of the biosurfactant produced with *P. mosselii* MP-6; Figure S7. Critical micelle concentration (CMC) of the biosurfactant produced with *P. mosselii* MP-6; Figure S8. Chemical structure of (a) NAP, (b) PHE, (c) PYR, and (d) BaP; Table S1. Results of the tests used for screening biosurfactant-producing strains.
