# Supplementary material for: Enhanced Solubilization and Biodegradation of HMW-PAHs in Water with a Pseudomonas mosselii-Released Biosurfactant
Source: Polymers (Basel). 2023 Nov 29;15(23):4571. doi: 10.3390/polym15234571 (PMC10708242; doi:10.3390/polym15234571)
Supplement: Supplementary file 1 [file polymers-15-04571-s001.zip › polymers-2705090-supplementary.pdf]

Supporting Information for

Enhanced Solubilization and Biodegradation of HMW-PAHs in Water with a *Pseudomonas mosselii*-Released Biosurfactant

Mingqian Xia *, Shibin Wang, Bo Chen, Rongpeng Qiu and Gongduan Fan

College of Civil Engineering, Fuzhou University, Fuzhou 350116, China

* Correspondence: mq.xia@fzu.edu.cn

Text. S1. Screening for biosurfactant producing strains.

Text. S2. SEM observation of the cells.

Text. S3. Biosurfactant tolerance to extreme environmental conditions.

Text. S4. HPLC determination of PAHs.

Figure. S1. (a) Drop collapse results and (b) Emulsification stability (E24) test of the bacterial supernatants.

Figure. S2. Fermentation culture of *P. mosselii* MP-6 and extraction process of the biosurfactant.

Figure. S3. (a) Growth curve of *P. mosselii* MP-6 in LB broth under 30°C; (b) The relationship between optical density (OD_{600}) and total bacterial count of *P. mosselii* MP-6.

Figure. S4. The growth curves of *P. mosselii* MP-6 under different (a) pH; (b) NaCl and heavy metals concentrations: (c) Ni^{2+}; (d) Pb^{2+}; (e) Cd^{2+}; (f) Cr^{6+}.

Figure. S5. FTIR spectroscopy of the biosurfactant produced by *P. mosselii* MP-6.

Figure. S6. LC-MS results of the biosurfactant produced by *P. mosselii* MP-6.

Figure. S7. Critical micelle concentration (CMC) of biosurfactant produced by *P. mosselii* MP-6.

Figure. S8. Chemical structures of NAP, PHE, PYR and BaP.

Table S1. Results of the tests used for screening biosurfactant producing strains.

Text. S1. Screening for biosurfactant producing strains.

- Oil spreading assay

20 mL of deionized water was added to a 25 mL sterile culture dish (diameter 100 mm), then 10 μL crude oil was added to form a thin film on the surface of deionized water. 10 μL cell free supernatant was dropped at the center of the oil film, observing the area of the transparent circle at the center of the oil film. The larger the diameter of the circle, the higher the content and activity of the surfactant produced by the bacterial strain.

- Drop collapse assay

10 μL light paraffin oil was added to the cover of a 96 well plate, balance the plate at 37 °C for 1 hour, and then add 5 μL cell-free supernatant to the surface of the paraffin oil (add 5 μL of deionized water as the control). Observe the results in the plate holes after 1 minute. If the supernatant collapses on the oil surface, it was considered positive, indicating the presence of biosurfactant in the bacterial culture. Conversely, it was considered negative.

- Surface tension measurement

The hanging drop method was used to measure the surface tension of the bacterial culture. 10 μL culture supernatant was taken to measure surface tension using the contact angle measuring instrument JC200D4 (deionized water was used as the control). The lower the surface tension of the liquid, the higher the content and activity of surfactants produced by the bacteria.

- Emulsification stability (E24) measurement

The emulsification index (E24) was determined by mixing an equal volume of hydrophobic substance (gasoline) and cell-free supernatant of the strain. Rotate the two incompatible mixtures at high speed on a vortex for 1 min, let them stand at room temperature for 24 h, and measure the height of the emulsion layer. The calculation formula for emulsification index E24 is shown below:

$$E24 = \frac{H_0}{H_t} \times 100\%$$

In the formula, E24 represents the emulsification index of the mixed solution after 24 hours of standing, H_0 represents the height of the emulsification layer (cm), and H_t represents the total height of the liquid (cm).

Text. S2. SEM observation of the cells

The steps for scanning electron microscopy (SEM) observation of bacterial cells morphology are as follows:

1) Inoculate the bacterial strain to LB broth and culture for 12 h at 30°C, 130 rpm. Transfer 8 mL of the bacterial culture to a sterile 10 mL centrifuge tube.

2) Centrifuge the tube at a speed of 4500 rpm for 15 min, discard the supernatant, and add 2.5% glutaraldehyde, avoiding light. Fix the cells by refrigerating at 4°C for 2 h.

3) After refrigeration, centrifuge the sample at a speed of 4500 rpm for 15 minutes, discard the supernatant, and wash three times with 0.1 mol/L PBS buffer (at 4500 rpm for 15 minutes each time) to remove residual glutaraldehyde.

4) Prepare ethanol solutions with concentrations of 30%, 50%, 70%, 80%, 90%, and 100% for gradient dehydration. Ethanol dehydration steps: Add ethanol of different concentrations to the bacterial pellet, gently shake to suspend, and then let it settle. Centrifuge (at 4500 rpm for 15 minutes) and discard the supernatant. Repeat the addition of ethanol at different concentrations for gradient dehydration, let it settle for 5-7 minutes each time before centrifugation. Finally, dehydrate twice with 100% ethanol, let it settle for 5-7 minutes each time before centrifugation.

5) Prepare tert-butanol solutions with concentrations of 50%, 70%, 85%, 95%, and 100% for substitution. Tert-butanol substitution steps: Add tert-butanol of different concentrations to the bacterial pellet, gently shake to suspend, and then let it settle. Centrifuge (at 4500 rpm for 15 minutes) and discard the supernatant. Repeat the addition of tert-butanol at different concentrations for substitution, let it settle for 5-7 min each time before centrifugation. Subsequently, perform 2 substitutions with 100% tert-butanol, let it settle for 5-7 minutes each time before centrifugation, and discard the supernatant.

6) Finally, add a small amount of 100% tert-butanol to submerge the pellet.

7) After vertexing and mixing, drop the suspended bacterial liquid onto a single crystal silicon wafer and let it dry overnight at room temperature. Before testing, coat the silicon wafer with gold and use the ultra-high-resolution field emission scanning electron microscope (Verios G4 model) from Thermo Fisher Scientific to observe the bacterial morphology.

Text. S3. Biosurfactant tolerance to extreme environmental conditions.

- Temperature

The cell-free supernatant was incubated at different temperatures for 2 hours, with the following temperature ranges: 4, 10, 20, 30, 40, 50, 60, 70, 80, 90, and 100°C. After incubation, the supernatants were cooled to room temperature and left to stand for 24 hours. The emulsifying index E24 and surface tension were measured at each temperature to determine the biosurfactant's tolerance to different temperature stresses.

- NaCl concentrations

To obtain cell-free supernatant with different salt concentrations, different amounts of NaCl were added to the cell-free supernatant. The NaCl gradient was set as follows: 0, 10, 20, 30, 40, 50, 60, 80, and 100 g/L. After adding NaCl, the cell-free supernatants were left to stand at room temperature for 24 hours. The emulsifying index E24 and surface tension were measured at different NaCl concentrations to determine the biosurfactant's tolerance to NaCl (0-100 g/L) stresses.

- pH

The pH of the cell-free supernatant was adjusted to 4, 5, 6, 7, 8, 9, and 10 using 1 mol/L HCl/NaOH solutions. The supernatants at different pH values were left to stand for 24 hours. The emulsifying index E24 and surface tension of the cell-free supernatant at different pH levels were measured to determine the biosurfactant's tolerance to pH stresses.

- Heavy metals

To obtain cell-free supernatant with different concentrations of heavy metals, $NiCl_2$, Cd $(NO_3)_2$, $K_2Cr_2O_7$, and $Pb(NO_3)_2$ were added to the supernatant. The ion concentration gradients were set as follows: 0, 10, 20, 30, 40, 50, 60, 80, and 100 mg/L. After adding heavy metals, the cell-free supernatants were left to stand at room temperature for 24 hours. The emulsifying index E24 and surface tension were measured at different concentrations of Ni^{2+}, Cd^{2+}, Pb^{2+}, and Cr^{6+} to determine the tolerance of the biosurfactant to heavy metals (0-100 mg/L) stresses.

Text S4. HPLC determination of PAHs.

To determine the dissolved PAHs, an equal amount of chromatography grade n-hexane was added to extract the organic compounds. The extraction process was repeated three times, and all organic phases were collected. The collected samples were then dehydrated and concentrated to dryness with anhydrous sodium sulfate and redissolved in chromatography-grade methanol. 1 mL aliquot of the solution was filtered through a 0.22 μm syringe filter (nylon, organic series) and stored in a 2 mL brown HPLC vial, which was then brought to a final volume of 1 mL. All samples were stored in a refrigerator at 4°C for HPLC detection. The concentrations of NAP, PHE, PYR, and BaP in all degradation experiments were quantitatively analyzed using HPLC (Shimadzu LC-2030, Agilent XDB-C18, 4.6 mm × 250 mm, 5 μm). The detection conditions were as follows: the mobile phase was methanol/water (90:10), flow rate was 1 mL/min, column temperature was set at 35°C, UV detection wavelength was 254 nm, and each injection volume was 20 μL.

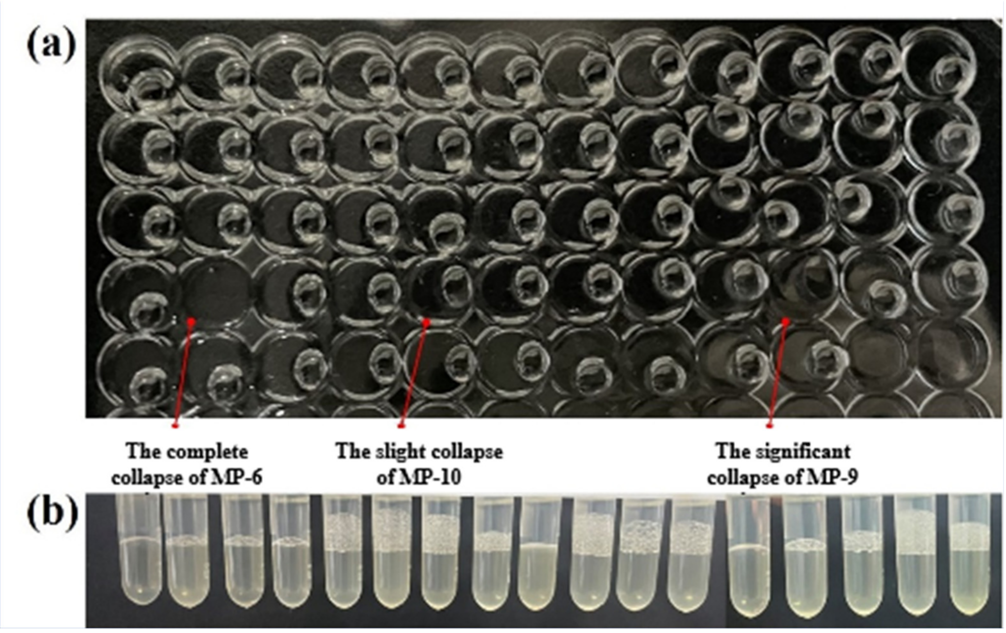

Figure. S1 (a) Drop collapse results and (b) Emulsification stability (E24) test of the bacterial supernatants.

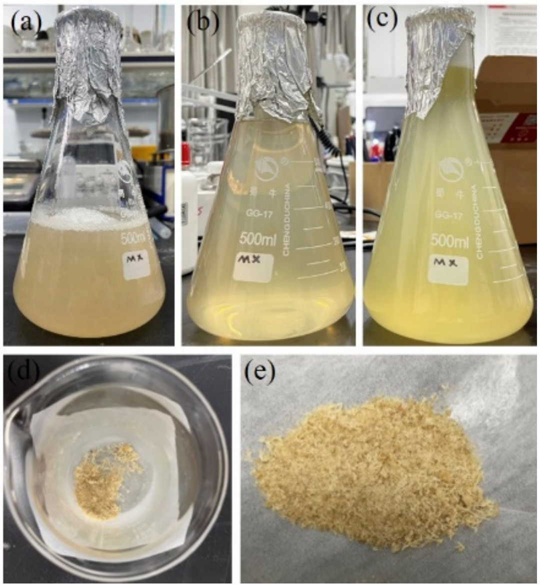

Figure. S2 Fermentation culture of *P. mosselii* MP-6 and extraction process of the biosurfactant.

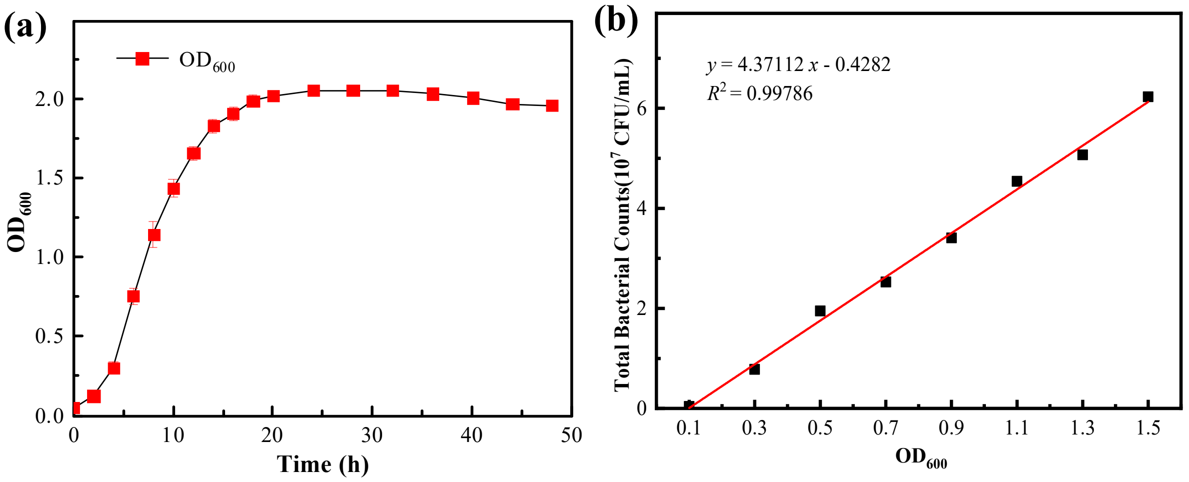

Figure. S3 (a) Growth curve of *P. mosselii* MP-6 in LB broth under 30°C; (b) The relationship between optical density (OD_{600}) and total bacterial count of *P. mosselii* MP-6.

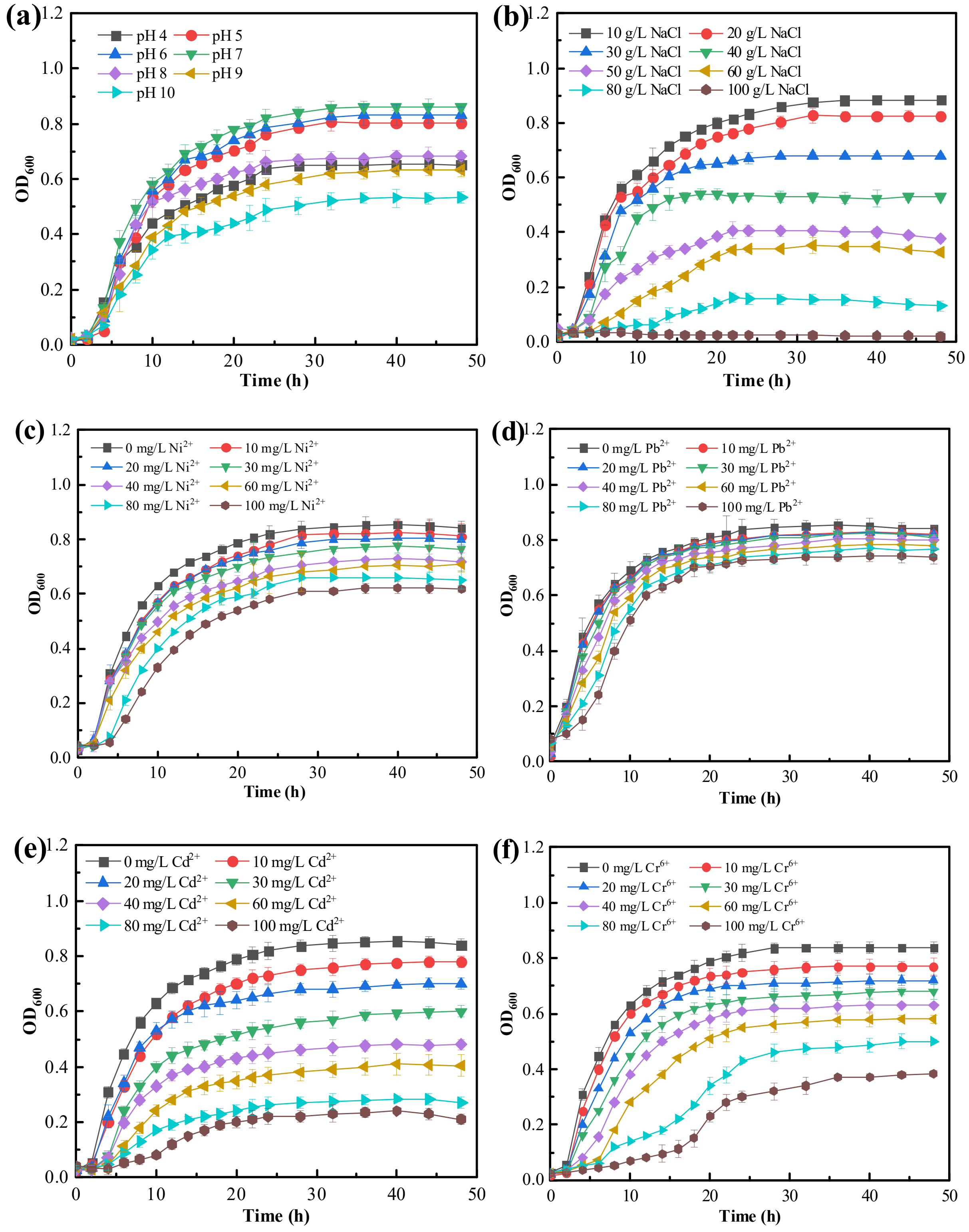

Figure. S4 The growth curves of *P. mosselii* MP-6 under different (a) pH; (b) NaCl and heavy metals concentrations: (c) Ni^{2+}; (d) Pb^{2+}; (e) Cd^{2+}; (f) Cr^{6+}.

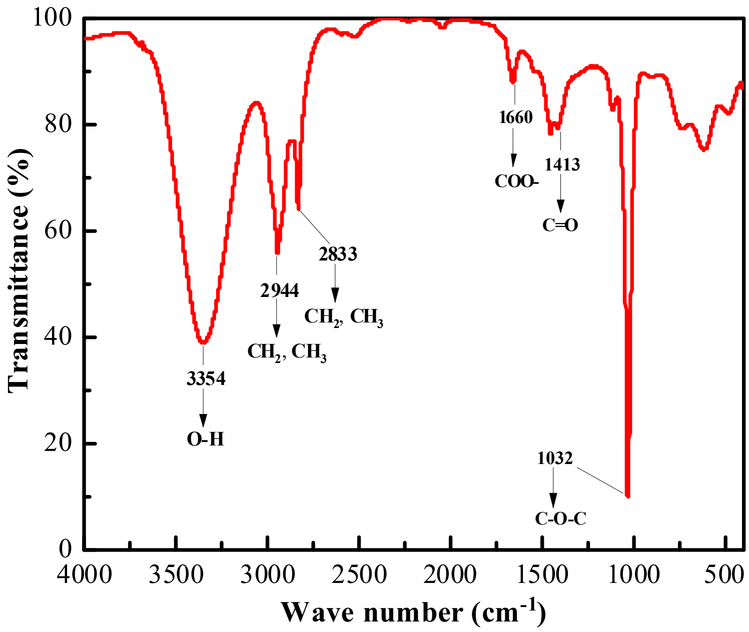

Figure. S5 FTIR spectroscopy of the biosurfactant produced by *P. mosselii* MP-6.

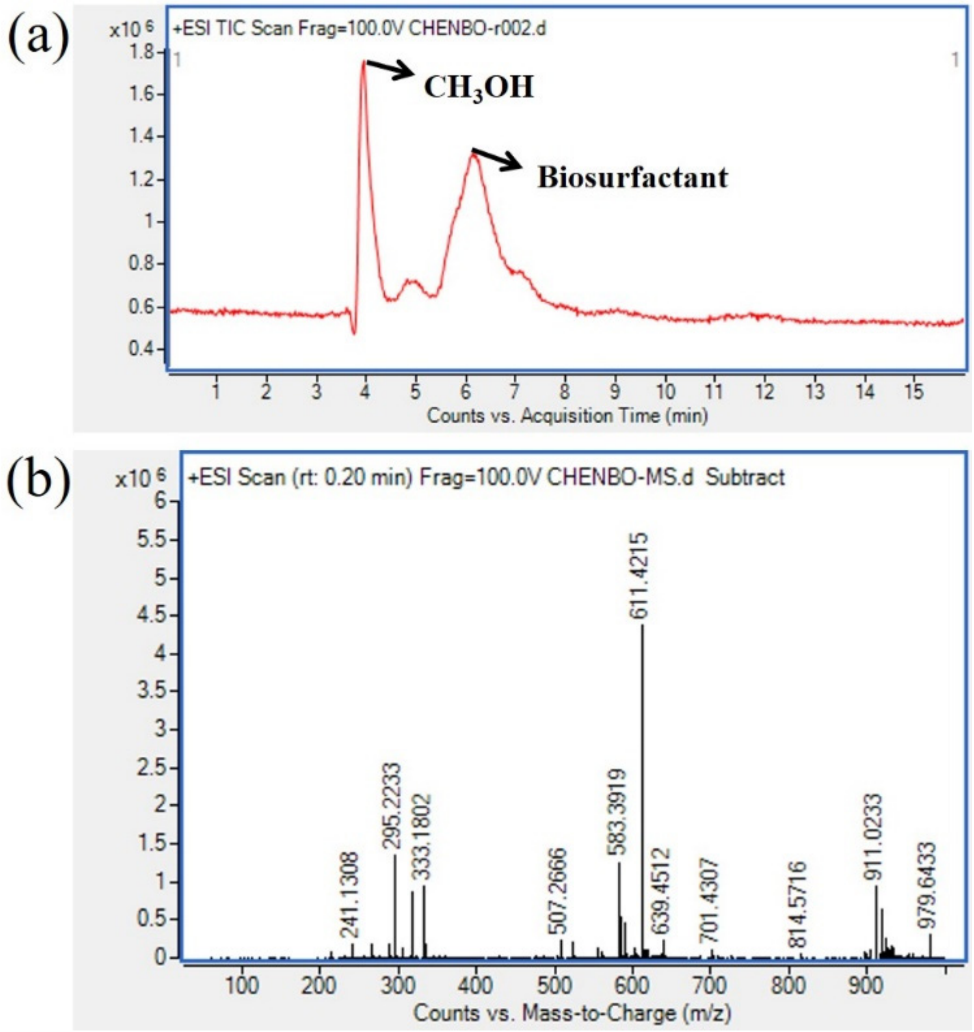

Figure. S6 LC-MS results of the biosurfactant produced by *P. mosselii* MP-6.

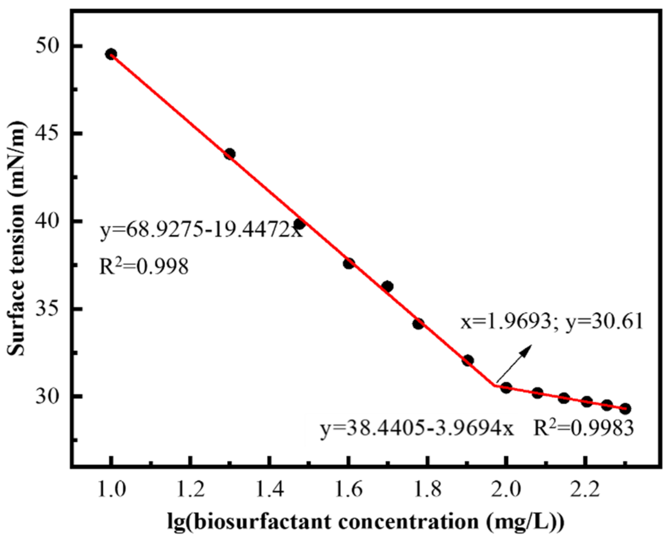

Figure. S7 Critical micelle concentration (CMC) of biosurfactant produced by *P. mosselii* MP-6.

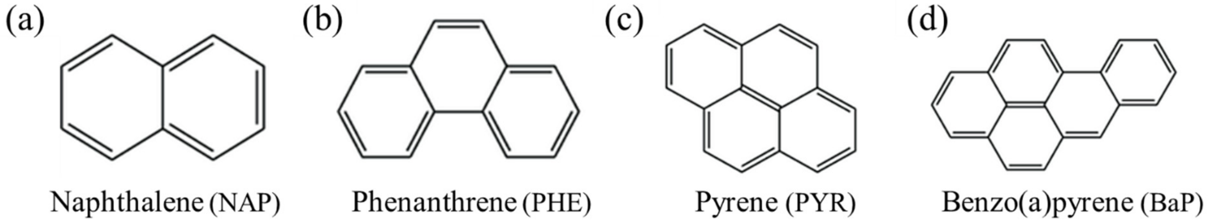

Figure. S8 Chemical structures of (a) NAP; (b) PHE; (c) PYR and (d) BaP.

Table S1 Results of the tests used for screening biosurfactant producing strains.

Strains	Droplet collapse method	Oil drain ring diameter（mm）	E24（%）	Surface tension（mN/m）
MP-1	Not collapsing	8.9±0.5	2.2±0.4	56.0±0.5
MP-2	Not collapsing	0.0±0.0	20.3±1.5	65.4±0.8
MP-3	Not collapsing	3.5±0.3	28.6±0.6	70.2±0.4
MP-4	Not collapsing	0.0±0.0	16.5±1.1	71.3±0.2
MP-5	Not collapsing	0.0±0.0	4.4±2.4	68.0±0.5
**MP-6**	**Complete collapse**	**21.3±1.1**	25.1±2.5	**34.3±0.8**
MP-7	Not collapsing	2.8±0.5	15.4±1.2	71.3±0.5
MP-8	Not collapsing	10.4±0.5	22.2±2.3	52.2±1.1
MP-9	Partial collapse	3.3±0.1	12.4±1.1	48.2±0.3
MP-10	Weak collapse	7.7±0.4	0.0±0.0	46.6±0.5
MP-11	Not collapsing	1.1±0.2	0.0±0.0	62.8±0.3
MP-12	Not collapsing	2.3±0.1	30.4±2.1	66.4±0.6
MP-13	Not collapsing	4.4±0.2	21.1±1.1	60.8±0.1
MP-14	Not collapsing	0.0±0.0	24.8±2.3	61.7±0.3
